# Psychological, social and technical factors influencing electronic medical records systems adoption by United States physicians: a systematic model

**DOI:** 10.1186/s12961-022-00851-0

**Published:** 2022-05-02

**Authors:** Raghid El-Yafouri, Leslie Klieb, Valérie Sabatier

**Affiliations:** 1 Grenoble Ecole de Management, Grenoble, France; 2 Tenxor Inc, San Francisco, CA USA

**Keywords:** Electronic medical records adoption, Electronic health records adoption, Innovation adoption, Technology acceptance, Intentions of physicians, Influence of policy

## Abstract

**Background:**

Wide adoption of electronic medical records (EMR) systems in the United States can lead to better-quality medical care at lower cost. Despite the laws and financial subsidies by the United States government for service providers and suppliers, interoperability still lags. An understanding of the drivers of EMR adoption for physicians and the role of policy-making can translate into increased adoption and enhanced information sharing between medical care providers.

**Methods:**

Physicians across the United States were surveyed to gather primary data on their psychological, social and technical perceptions towards EMR systems. This quantitative study builds on the theory of planned behaviour, the technology acceptance model and the diffusion of innovation theory to propose, test and validate an innovation adoption model for the healthcare industry. A total of 382 responses were collected, and data were analysed via linear regression to uncover the effects of 12 variables on the intention to adopt EMR systems.

**Results:**

Regression model testing uncovered that government policy-making or mandates and other social factors have little or negligible effect on physicians’ intention to adopt an innovation. Rather, physicians are directly driven by their attitudes and ability to control, and indirectly motivated by their knowledge of the innovation, the financial ability to acquire the system, the holistic benefits to their industry and the relative advancement of the system compared to others.

**Conclusions:**

Identifying physicians’ needs regarding EMR systems and providing programmes that meet them can increase the potential for reaching the goal of nationwide interoperable medical records. Government, healthcare associations and EMR system vendors can benefit from our findings by working towards increasing physicians’ knowledge of the proposed innovation, socializing how medical care providers and the overall industry can benefit from EMR system adoption, and solving for the financial burden of system implementation and sustainment.

**Supplementary Information:**

The online version contains supplementary material available at 10.1186/s12961-022-00851-0.

## Background

The United States relies on the national diffusion of electronic medical records (EMR) systems to benefit from efficiencies that can both reduce healthcare costs and improve quality of care. However, even with policy-making and substantial governmental financial investment, the feasibility of a national patient electronic health records system, backed by interoperability and data sharing with and between all medical practices, remains unproven. Since 1996, the United States government has supported the wider adoption of health information technology and electronic medical data exchange. The Health Insurance Portability and Accountability Act of 1996 established national standards for electronic healthcare transactions and national identifiers for healthcare providers. The American Recovery and Reinvestment Act (ARRA) of 2009, a stimulus package also known as the Recovery Act, allotted over $19 billion out of $145 billion for healthcare spending to modernize health information technology systems. Since 2009, the adoption of certified EMR systems that meet meaningful user criteria has seen a dramatic increase according to the Centers for Medicare & Medicaid Services (CMS). However, interoperability and health information exchange are problematic and have not reached their full potential [1, 2].

In this article, the term adoption will mean either implementing an EMR system for the first time or upgrading the current EMR system by introducing advanced capabilities. The anticipated benefits of EMR system adoption include real-time data access, clinical decision support, enhanced monitoring, computerized medication orders and simplified administrative and billing work [3, 4]. Advanced adoption and a wider spread of EMR systems can mean better medical care for the patient [5]. Efforts have been made by researchers to understand the drivers of and barriers to adoption [6–9]. What is still missing is a comprehensive model that compares different categories of factors so efforts can be focused on those that make the greatest impact on adoption.

The innovation implementation process in the United States healthcare industry is lengthy and complex, requiring multistage system setup and adoption [2, 10, 11]. The industry is composed of sponsors and providers in both the public and private sector. All bear responsibility for EMR system development and advancement. As a public healthcare sponsor, the United States government supports the adoption of EMR technologies to achieve cost and quality benefits and has achieved a reduction in Medicare expenditures [12]. However, the intentions of medical care providers have not been clear. Physicians and clinicians have divided opinions regarding EMR system advantages [13], and there is no consensus on how to achieve EMR system benefits across the United States healthcare system as a whole [9]. Furthermore, and contrary to the government’s expectations, convenient access to tests, including electronic imaging results, seems to encourage physicians to increase their ordering of testing and imaging, rather than reduce it [14].

System adoption and implementation has been easier at larger institutions than at smaller practices, in particular solo practices and non-primary care specialties [15], due to the greater access to resources and management capacity by larger entities [11, 16]. Physicians, whether they are part of larger institutions like hospitals or in smaller practices, make up a large EMR system user group [17], and their attitudes and perceptions shape long-term system success. Therefore, we pose the following research question: What factors influence physicians to adopt innovations such as EMR systems? And what impact do government policy and mandate have on the adoption?

Identifying the influence and interaction between the psychological, social and technical perspectives is required in order to understand the deep dynamics of an innovation and, in turn, measure its success [18, 19]. To investigate our research question, we researched the theoretical foundation of behavioural and technology acceptance theories, constructed a model, formed hypotheses, and invited physicians around the United States to participate in a quantitative Qualtrics survey (“Methods” section). In the “Results” section, we report the results and note which hypotheses were supported. In the “Discussion” section, we discuss our key findings. Finally, in the “Conclusions” section, we present our conclusions and highlight the practical implications of the findings. In our conclusions, we agree with Hung et al. [20] that physicians are emotionally driven by the extent to which EMR systems can deliver better healthcare service and conditions, and we further conclude that social factors in general, including government directives, have little effect. In turn, we suggest the need to develop educational programmes to increase system knowledge, clarify industry benefits, pinpoint relative advancement and facilitate financial support to drive positive attitudes and perceived behavioural control among physicians.

## Methods

### The behaviour of adoption

Rogers’s diffusion of innovation (DOI) theory [21], Davis’s technology acceptance model (TAM) [22] and Ajzen’s theory of planned behaviour (TPB) [23] are key technology adoption and behavioural theories to employ. Combining more than one theoretical model usually leads to a better understanding of an adoption phenomenon [24]. Thus, our model for EMR innovation adoption integrates technical, social, human and psychological factors from different theories and models, which is in line with what Jian et al. [25] did for uncovering EMR adoption factors in Taiwan. Our research question focuses on identifying the drivers behind physicians’ adoption of an innovation. The DOI theory [21] describes the spread of technical innovations, such as EMR systems, through an innovation-decision process [21]. This process is a series of stages that a decision-maker follows to reach a decision that can be favourable (to adopt) or unfavourable (to reject).

The decision for EMR system adoption can be modelled as a rational behaviour in the framework of the TPB [23]. The rational planned behaviour is mainly a function of the individual’s intention to engage in the behaviour, which is the indication of an individual’s readiness to perform the behaviour. The TPB variables can “capture unique variance in intention” (p. 178) [26]. Although the individual’s intention to act is not actual behaviour, “there is considerable evidence that intention to perform a behavior predicts actual behavior” (p. 174) [26].

### Intention drivers

Per the TPB model [23], the intention to engage in a certain rational behaviour is directly influenced by the attitude towards the behaviour, the subjective norms and the perceived behavioural control. Attitude is the individual’s mental state, including feelings, values and dispositions towards the behaviour, and subjective norms are the collection of social pressures and beliefs that important others (e.g. peers or government) expect for a particular behaviour [23]. Perceived behavioural control corresponds to the self-efficacy theory developed by Bandura [27], who defined it as the conviction by someone about his or her ability to successfully execute a behaviour required to produce the expected result.

Ajzen [23] noted that the TPB is intended to provide a general guideline of what determines or influences a behaviour. The researcher is expected to creatively identify the main factors affecting a behaviour that are relevant to a situation or setting. Hypotheses 1a and 1b are two of the hypotheses on predicting intention:

#### Hypothesis 1a

***Attitude*** *towards adoption has an effect on the* ***intention*** *to adopt.*

#### Hypothesis 1b

***Perceived behavioural control*** *over adoption has an effect on the* ***intention*** *to adopt.*

### Subjective norms factors

Subjective norms’ social pressures can be coercive, mimetic or normative [24]. Coercive pressure is practised by a source of power to force conformity to demand or expectations; mimetic pressure is what makes an individual imitate others; and normative pressure is the tendency to behave in a manner that is deemed to be acceptable or approved by others [23, 24]. Social interferences are generally expected to play a positive role during and after the adoption of a new technology [28, 29]. Therefore, identifying the factors of the subjective norms helps to assess their impacts on the intention to adopt EMR.

The medical industry has tight networks that provide forums for peer knowledge and opinion sharing. Peer preference is the individual’s perception of what medical peers, colleagues and associates think about EMR systems. Having similar or congruent perspectives among physicians and associating with medical care professionals is an important factor of behaviour [11]. According to Bramble et al. [30], physicians who know other physicians supporting EMR systems have a greater desire to adopt these systems themselves. Peer preference exerts a mimetic pressure and is a main component of social norms. Hypothesis 1c is an additional hypothesis on intention:

#### Hypothesis 1c

***Peer preference*** *has an effect on the* ***intention*** *to adopt.*

According to Watkins et al. [31], governments have been known to “play a central orchestrating role in the generation and diffusion of innovation in a national economy” (p. 1408). This orchestrating role is clearly visible in the United States government’s regulations, policies and mandate requiring the adoption and use of EMR systems. This exerts coercive pressure on physicians who feel that their decisions should meet the expectations of the government and other influential organizations [24]. Hence, hypothesis 1d is proposed:

#### Hypothesis 1d

*The* ***government policy and mandate*** *for adoption have an effect on the* ***intention*** *to adopt.*

More recent literature shows that intermediary industry associations have an increasing involvement in cooperative relations between government and industry aimed at influencing an innovation’s diffusion, adoption, training and standards [31]. These associations create and set industry protocols and common best practices to which physicians are driven to adhere. Lack of standardization is the greatest challenge according to Rathert et al. [2]. We expect physicians to take the normative pressure of meeting industry standards into consideration when adopting technologies, and this is reflected in hypothesis 1e:

#### Hypothesis 1e

***Industry standards*** *for adoption have an effect on the* ***intention*** *to adopt.*

### Attitude drivers

Hypothesis 1a posits that attitude has an effect on intention. In this section, factors that influence attitude are considered. Complex innovation integration, like EMR systems, requires knowledge creation and diffusion [18, 32]. Knowledge is the extent to which the individual is aware of the innovation and its purpose, structure, components, requirements, benefits and impacts. According to Rogers [21], knowledge is the first stage of the innovation-decision process by which a decision regarding the adoption of innovation matures prior to being made. Knowledge leads to physicians being persuaded to adopt the system [21].

Having such knowledge helps promote persuasion and attitude formation. This definition supports hypothesis 2a:

#### Hypothesis 2a

***Knowledge*** *of innovation has an effect on the* ***attitude*** *towards adoption.*

Ultimately, successful adoption of EMR systems in the United States and the nationwide diffusion of shareable electronic records should yield measurable benefits. Several studies associate the acquisition and adoption of technology innovation with enhanced performance, increased efficiencies and improved quality [9, 33, 34]. The more the adopters believe they can achieve higher performance, efficacies and quality, the more they will have positive psychological feelings towards it. The TAM [22] posits two determinants of users’ attitude towards acceptance of a technology system: perceived usefulness and perceived ease of use. Davis [22] defined perceived usefulness as the extent to which a person believes that using a system will enhance the individual’s job performance. However, regarding benefits, an innovation can differ in its usefulness to the user and to the overall industry. Thus, a distinction is needed between perceived usefulness for the individual and perceived benefits for the industry. While perceived usefulness is a behavioural belief, perceived industry benefits are an outcome evaluation [26]. Both are factors of attitude [26]. Hence, hypotheses 2b and 2c are as follows:

#### Hypothesis 2b

***Perceived industry benefits*** *have an effect on the* ***attitude*** *towards adoption.*

#### Hypothesis 2c

***Perceived usefulness*** *has an effect on the* ***attitude*** *towards adoption.*

Perceived ease of use, the second determinant of technology acceptance, is defined by Davis [22] as the degree to which a person believes using the system would be free of effort. Perceived ease of use can influence attitude and is stated in hypothesis 2d:

#### Hypothesis 2d

***Perceived ease of use*** *has an effect on the* ***attitude*** *towards adoption.*

### Perceived behavioural control drivers

Researchers such as Boonstra and Broekhuis [35], Bramble et al. [30], Felt-Lisk et al. [3], Häyrinen et al. [36], Hillestad et al. [4] and Mostashari et al. [37] have discussed and identified existing and potential control barriers to EMR system adoption. The two main factors identified are, first, not having the financial ability for adoption, and second, the negative impact of EMR system adoption on workflow and operations. Financial ability is having the necessary funds to support the initial setup and ongoing maintenance of the system. The impact on workflow is the extent to which the EMR system benefits or disrupts operations. These factors are what Ajzen [23] calls control beliefs. They affect behaviour through their impact on the trust in self and the perception that one is feasibly able to adopt and use an innovation. Two of the hypotheses of factors impacting perceived behavioural control are as follows (hypotheses 3a and 3b):

#### Hypothesis 3a

***Financial ability*** *to adopt has an effect on the* ***perceived behavioural control*** *over adoption.*

#### Hypothesis 3b

***Workflow benefits*** *from adoption have an effect on the* ***perceived behavioural control*** *over adoption.*

Additionally, understanding how much exposure physicians have to EMR systems may help to assess the role in their willingness to adopt and use the system, especially when this adoption comes with a financial or patient-care-related advantage over other competing offices. Also, there are early adopters [21] who jump on opportunities to use an innovation before others in order to differentiate themselves. Relative advancement is the perception of how much more or less current the setup at the physician’s facility is compared with others. It is also related to the trialability and observability attributes of Rogers’s DOI theory [21], where people’s willingness to adopt an innovation is greater if they had the opportunity to experience it; thus, we propose hypothesis 3c:

#### Hypothesis 3c

***Relative advancement*** *of adoption has an effect on the* ***perceived behavioural control*** *over adoption.*

Finally, Ajzen [23] showed that attitude and perceived behavioural control have respective effects on each other. Therefore, we hypothesize (hypotheses 2e and 3d) that our results will show:

#### Hypothesis 2e

***Perceived behavioural control*** *over adoption has an effect on the* ***attitude*** *towards adoption.*

#### Hypothesis 3d

***Attitude*** *towards adoption has an effect on* ***perceived behavioural control*** *over adoption.*

**Fig. 1:**
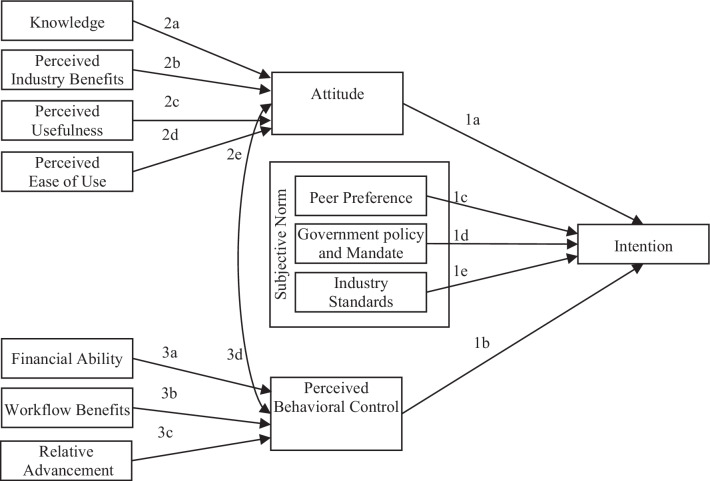
Conceptual EMR adoption model and hypotheses—diagram of the hypotheses to be tested, where the boxes are the variables and the arrows are the directional effect of one variable on the other

Figure 1 illustrates the constructs, the hypotheses and the overall model to be tested. The model combines the TAM and the TPB. Both are a specialization and derivation of the theory of reasoned action (TRA) [38]. Merging of the TPB and the TAM combines compatible models and is therefore natural and possible. The predictive power of both the TAM and the TPB is empirically about the same [26], so that cannot guide the modelling. It is possible to use the TAM constructs perception of usefulness and ease of use as direct antecedents of intention to implement a higher level of EMR, or to use the TPB for that goal. Both have been done in the literature. The TPB is preferred here because industry-wide outcome evaluations, like benefits for the whole industry, are a part of attitude in the TPB. This and behavioural control, a natural and direct part of the TPB but not of the TAM, are necessary to understand the reason behind the intentions of the decision-makers. Using the TPB as basis is therefore “the more suitable theoretical framework” (p. 961) [38] because it leads to a deeper understanding of the voluntariness of the decision-maker. These considerations lead directly to the model of Fig. 1.

### Survey creation and distribution

A structured survey questionnaire targeting a large group of physicians was developed for the purpose of this study. The final survey questions were prepared to operationalize the variables and test the hypotheses shown in Fig. 1. There were 13 variables (see Table 1). Responses to the questions were collected using Likert scales. Invitations to complete the survey were sent via direct mail, email and social media advertising. A total of 2012 mailings were sent to a network of Michigan physicians who were associated with groups including Beaumont Hospital and Henry Ford Health Systems. In addition, 55,177 emails were sent to selected groups of physicians nationwide. Facebook and LinkedIn advertisements reached 5978 physicians specifically by using the segmentation and audience targeting tools offered by both social platforms. Although three methods were used to disseminate the invitation, the survey itself was distributed digitally using Qualtrics software and was the same for all respondents. An easy-to-access web address (www.adoptingEMR.com) was shared with invitees to link them to the survey. The distribution and collection of answers took approximately 10 weeks.

**Table 1:** Variables and their descriptive statistics

Variable	No.	Mean	SD	SEM
Intention	208	5.05	1.830	0.127
Attitude	230	5.13	1.876	0.124
Perceived behavioural control	230	4.74	1.781	0.117
Peer preference	220	4.40	1.678	0.113
Government policy and mandate	343	4.99	1.855	0.100
Industry standards	343	4.46	1.791	0.097
Knowledge	378	3.62	0.945	0.049
Perceived industry benefits	360	3.54	1.814	0.096
Perceived usefulness	334	4.08	2.040	0.112
Perceived ease of use	332	3.60	1.710	0.094
Financial ability	217	4.21	2.054	0.139
Workflow benefits	331	3.25	1.737	0.095
Relative advancement	342	5.25	1.329	0.072

The constructs of the conceptual model and their descriptive statistics of the mean, standard deviation (SD) and standard error of the mean (SEM). Responses were gathered on a Likert scale (1 = completely negative, 7 = completely positive), except for knowledge (1 = not at all knowledgeable, 5 = completely knowledgeable). *No.* = total responses received for that question

The study was performed in accordance with the Rules and Regulations of Grenoble Ecole de Management (GEM) Doctoral School (Grenoble, France) and was approved by GEM’s Ethics Review Board, which adopts the Academy of Management (AOM) Code of Ethics. The invitation included a clear description of the purpose of the study, and all participants consented digitally to the use of the data and results for academic and scholarly purposes as a condition for entering the Qualtrics online survey.

## Results

### Response conversion and distribution

The email campaigns achieved a 12% opening rate (6690 out of 55,177). Of the 6690 people who opened their emails, 437 clicked on the survey, for a conversion rate of 7%. Of the 5978 people who viewed the social media ads, 143 clicked to access the survey, for a conversion rate of 2%. The conversion rate for direct mail could not be measured, but assuming an industry rate of 3.5%, 70 people would have visited the survey from direct mail. Therefore, a total of 650 (437+143+70) people overall opened the survey. The final survey was completed by 382 participants (59% of those who visited it)—a sample size that meets and exceeds the need to conduct multivariate analysis for our 13 items [39]. Only 43 people (11%) left one or more questions unanswered.

Of the 382 respondents, 271 (71%) were male and 111 (29%) were female physicians. Sixty-two physicians (16%) were younger than 45 years of age, 127 (33%) were between 45 and 54 years old, 128 (34%) were between 55 and 64 years old, and 65 (17%) were at least 65 years old. They were spread across 47 different US states, with the following states accounting for 50% (191) of the entries: Michigan, California, New York, Texas, Pennsylvania, Ohio, Florida, Maryland and Washington. The largest group of 136 (36%) had 20–29 years of experience, followed by 123 (32%) with 30 years or more of experience. Ninety-one (24%) of the physician participants had 10–19 years of experience, while 30 (7%) had less than 10 years of experience. Two participants did not specify years of experience. Specialist physicians were the largest group at 215 (56%), 64 were primary care doctors (17%), another 64 (17%) were surgeons, 25 (6%) were hospitalists or in emergency care, and 14 (4%) did not specify the practice type. Regarding the practice structure, 119 (31%) physicians were part of private offices with fewer than 10 physicians, 65 (17%) were part of private offices with 10 or more physicians, 131 (34%) were members of hospital-owned practices, 23 (6%) were part of government-owned practices, and 44 (12%) did not specify. Of the 382 physician respondents, 127 (33%) were solo or part owners of their practice, 224 (59%) were employees, 18 (5%) were contractors and 13 (3%) did not specify their employment type.

Prior to the analysis of the hypotheses, we ran correlation and regression analyses with all the demographic variables as independent variables. None of the demographic variables (age, gender, location, years of experience, practice type, practice structure and employment type) had any significant relationship with any of our dependent variables: intention, attitude and perceived behavioural control. Specifically, respondents came from a variety of states, but their location had no influence on the dependent variables used in this work. This set the stage for us to focus on the analysis of those variables that are represented in our hypotheses.

### Hypothesis and model testing

**Fig. 2:**
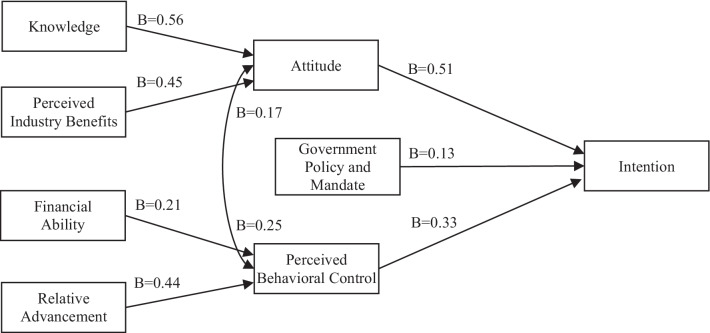
Final EMR adoption model verified by multiple regression and mediation tests. The direction of the arrows represents the effect of one variable over the other, and the *B* value is the strength of the effect. All coefficients are significant at *p* < 0.01

Descriptive statistics for the 13 variables are presented in Table 1. Three linear regression tests were run, one for each of the independent variables—intention, attitude and perceived behavioural control—as driven by the hypotheses of the conceptual model in Fig. 1. The Statistical Package for the Social Sciences (SPSS) was used and SAMPL [Statistical Analyses and Methods in the Published Literature] guidelines [40] were followed. We began by using the backward method for each regression, treating missing values using listwise deletion. Then, we ran forward and stepwise regressions, also with listwise deletion, to confirm that the solution with the highest *R*^2^ was found. The tests were two-tailed. The histogram and the P–P normal probability plots verified normal distribution for each test. The results of the three models, which represent 95% confidence intervals, are presented in Table 2, and Fig. 2 illustrates the significant relations (at *p* < 0.01 level) and their coefficients.

**Table 2:** Regression results of final EMR adoption models

Model	*R*^2^	*F*()	Predictors	*B*	*β*	*p*
Intention	0.56	*F*(3,185) = 77.18	Attitude	0.51	0.55	< 0.001
			Perceived behavioural control	0.33	0.32	< 0.001
			Government policy and mandate	0.13	0.14	0.007
Attitude	0.40	*F*(3,192) = 41.80	Knowledge	0.56	0.28	< 0.001
			Perceived industry benefits	0.45	0.46	< 0.001
			Perceived behavioural control	0.17	0.17	0.008
Perceived behavioural control	0.39	*F*(3,184) = 39.29	Financial ability	0.21	0.25	< 0.001
			Relative advancement	0.44	0.36	< 0.001
			Attitude	0.25	0.26	< 0.001

Summary of the results of the three linear regression tests showing the dependent variables, their predictors and the coefficients of the significant relations at *p* < 0.01 level

Five variables of attitude, perceived behavioural control, peer preference, government policy and mandate, and industry standards were used as independent variables to measure their impact on the dependent variable intention. Two of the five variables were removed: peer preference (*p* = 0.29) and industry standards (*p* = 0.13). A model with three significant relationships was reached explaining 55.6% of the variance (*R*^2^ = 0.56, *F*(3,185) = 77.18, *p* < 0.001). The three predictors of intention are attitude (*B* = 0.51, *p* < 0.001), perceived behavioural control (*B* = 0.33, *p* < 0.001), and government and mandate (*B* = 0.13, *p* = 0.007).

For predicting attitude, we began the regression testing with the following five independent variables based on the hypotheses in Fig. 1: knowledge, perceived industry benefits, perceived usefulness, perceived ease of use and perceived behavioural control. Two of the five variables, perceived usefulness (*p* = 0.16) and perceived ease of use (*p* = 0.47), were excluded as nonsignificant. Three predictors remained in the model, explaining 40% of attitude’s variance (*R*^2^ = 0.40, *F*(3,191) = 41.80, *p* < 0.001). Attitude can be significantly predicted by knowledge (*B* = 0.56, *p* < 0.001), perceived industry benefits (*B* = 0.45, *p* < 0.001) and perceived behavioural control (*B* = 0.17, *p* = 0.008).

Finally, perceived behavioural control is hypothesized (Fig. 1) to be affected by financial ability, workflow benefits, relative advancement and attitude. After entering those independent variables in the regression test, one of the four variables, workflow benefits (*p* = 0.67), was found nonsignificant. Three of the four predictors were significant and explained 39% of the variance of perceived behavioural control (*R*^2^ = 0.39, *F*(3,184) = 39.29, *p* < 0.001). Financial ability (*B* = 0.21, *p* < 0.001), relative advancement (*B* = 0.44, *p* < 0.001) and attitude (*B* = 0.25, *p* < 0.001) predicted perceived behavioural control.

### Model verification

Table 3 summarizes the results for the hypotheses. The final structural model in Fig. 2 represents nine significant relationships. The direct relations with intention support the TPB [23]; however, the relevance of this model is not its support of Ajzen’s TPB [23]. Rather, it is the presence of the variables of knowledge, perceived industry benefits, financial ability and relative advancement, and the strength of their impact on the constructs of intention, attitude and perceived behavioural control that make it valuable.

**Table 3:** Results for hypotheses

No.	Hypothesis	Supported
1a	**Attitude** towards adoption has an effect on the **intention** to adopt	Yes
1b	**Perceived behavioural control** over adoption has an effect on the **intention** to adopt	Yes
1c	**Peer preference** has an effect on the **intention** to adopt	No
1d	The **government policy and mandate** for adoption have an effect on the **intention** to adopt	Yes
1e	**Industry standards** for adoption have an effect on the **intention** to adopt	No
2a	**Knowledge** of innovation has an effect on the **attitude** towards adoption	Yes
2b	**Perceived industry benefits** have an effect on the **attitude** towards adoption	Yes
2c	**Perceived usefulness** has an effect on the **attitude** towards adoption	No
2d	**Perceived ease of use** has an effect on the **attitude** towards adoption	No
2e	**Perceived behavioural control** over adoption has an effect on the **attitude** towards adoption	Yes
3a	**Financial ability** to adopt has an effect on the **perceived behavioural control** over adoption	Yes
3b	**Workflow benefits** from adoption have an effect on the **perceived behavioural control** over adoption	No
3c	**Relative advancement** of adoption has an effect on the **perceived behavioural control** over adoption	Yes
3d	**Attitude** towards adoption has an effect on **perceived behavioural control** over adoption	Yes

Summary of the results for the 14 hypotheses shows that nine were supported and five were not

Following Baron and Kenny’s [41] method for mediation, we analysed three regression tests to verify the structure of the model. The first was whether the four independent variables of knowledge, perceived industry benefits, financial ability and relative advancements have direct effects on intention. The results of the first backward regression test did show a significant model (*R*^2^ = 0.28, *F*(3,170) = 21.79, *p* < 0.001) with three predictors after the exclusion of financial ability (*p* = 0.43). The second test was to verify whether the mediators of attitude and perceived behavioural control have a direct effect on intention. The model of the second test was significant (*R*^2^ = 0.55, *F*(2,203) = 123.54, *p* < 0.001), showing direct effects of attitude and perceived behavioural control on intention per the results in Table 2 after the exclusion of government mandate. Lastly, the third test was to determine whether the four independent variables and the mediators together predict intention. The results of the third test showed a significant model (*R*^2^ = 0.44, *F*(3,169) = 45.03, *p* < 0.001), with only attitude and perceived behavioural control as significant predictors of intention. All the effects of the other four independent variables dropped out, which validates the model in Fig. 2 and proves there is complete mediation by attitude and perceived behavioural control on the effects of knowledge, perceived industry benefits, financial ability and relative advancements on intention to adopt.

## Discussion

This research contributes to the research community by exposing the specific and unique drivers of healthcare providers’ behaviours: we built on the TPB [23], the TAM [22] and Rogers’s DOI theory [21], and proposed, tested and validated an EMR adoption model for measuring the intention to adopt innovation technology specifically in the healthcare industry.

We found that social factors have a very limited effect on the intention to adopt. Peer preference and industry standards show no effect, and the effect of government policy and mandate is negligible. It was initially expected that government laws and mandates, constituting its policy, would impose social pressure and would strongly affect the intention to adopt. However, this is not the case. The effect of government policy and mandate is the weakest of the three intention predictors (*β* = 0.14, Table 2). At the theoretical level, this result does not contradict the potential role played by governments as orchestrators of the generation of innovation in a national economy and the concept of the national innovation system, as described by Watkins et al. [31], but it does question the importance of the government’s role and its effectiveness in achieving results in DOI in settings comparable to national healthcare systems.

Attitude has the strongest effect on intention to adopt an innovation in healthcare (*β* = 0.55, Table 2), and it mediates the two effects of knowledge and perceived industry benefits. Physicians are highly concerned for the industry’s well-being and must witness tangible benefits before forming a favourable opinion regarding the innovation. Attitude is influenced by the outcome evaluation of the industry’s well-being. As for the role of knowledge, this argument is aligned theoretically with the many studies advocating the need for knowledge to diffuse innovation [18, 21, 32]. Our study empirically shows the effect of knowledge on attitude and intention and quantifies its strength.

Perceived behavioural control over adopting an innovation in healthcare by physicians has the second strongest effect on intention (*β* = 0.32, Table 2) and it is driven by financial ability and relative advancement. To have confidence in the innovation, physicians seek financial support and clarity around the value such investment has relative to their current approach and the differentiation from other practices.

In comparing the strength of the effects on intention, we learn that physicians do not respond favourably to pressures, whether they are coercive, mimetic or normative. Physicians seem to rise above influences from authoritative power and do not respond to peer pressure. They trust in their own judgement on how to act and behave when it comes to introducing innovations and information technologies in their practice. This judgement is the product of internally formed psychological beliefs and attitudes and rational assessment of the implementation feasibility. Their attitudes are built on the available knowledge and facts they have accumulated on the subject and its true benefits or perceived outcome evaluation of the healthcare industry. Also, they are keen on requiring a financial model capable of sustaining the monetary capital needed for the innovation, along with unbiased proof that the innovation can bring a clear advancement over the current state or other practices. All this presents difficulties for the government in carrying out its policies.

### Limitations

This research has some limitations. It focused on physicians, as they comprise a large actor group. However, using the same model framework, it is also possible to uncover the intentions, attitudes and adoption drivers for other key actors, such as nurses, medical administrators, insurance companies, pharmacists, vendors, providers and patients, in EMR system adoption. This is left for future research.

Another limitation is that the study assumed that physicians’ offices and practices either do not have any form of EMR system adoption or that they are undergoing, or need to undergo, an advancement of their adoption. It did not include the drivers and interests of those who have achieved full adoption, nor is full adoption truly defined. For those respondents who noted that they worked in an environment where full adoption was already achieved, we cannot know what future development of EMR systems will be, and therefore we do not know how attitudes and intentions would impact the adoption of such hypothetical future systems. Drivers and behaviours at that stage may include satisfaction and adoption sustainment factors, and these can very well differ from those at prior levels of adoption.

The sampling methodology that is used in this work is well-known and used in all quantitative areas of business research. It effectively elucidates and establishes relatively strong multivariate relationships between variables, such as whether an increase in an aspect of attitude tends to make a difference in one's intention to upgrade, depending on the significance of the relationship. The sampling methodology is not able to answer questions such as “what percentage of doctors are interested in upgrading" or similar univariate questions.” Such questions are also not part of the research question of this work. Our hope is to motivate future research to uncover the intentions and adoption drivers of more pockets of United States physicians leveraging the same model.

Finally, our study focuses on physicians in the United States, where the healthcare system structure has a unique balance and combination between the government, public medical care, private insurance companies, and private healthcare providers. Other countries have different structures for healthcare whose impact on intentions and attitudes might be different and research seems to be less well developed.

## Conclusions

While the United States government has done a commendable job promoting and facilitating the setup and implementation of EMR systems in hospitals, laboratories and physician offices, taking those systems to a state where they are interconnected and interoperable across facilities, institutions, networks, regions and states requires further work. Understanding and confirming the EMR systems’ features to meet and service physicians’ primary needs and expectations of the systems can expedite the advancement of the systems into a holistic network of healthcare records accessible from anywhere by any medical partner, of course respecting privacy considerations. Our model and its results discussed here can serve as a guideline and template of the needs and drivers for physicians’ adoption and use.

Collaboration between payers and providers, in the public and private sector, is needed to encourage nationwide EMR system adoption. Specific roles and responsibilities must be identified and defined, and then each group should be made aware of and experience the tangible benefits from the adoption of technology in healthcare. A proven balance between medical expenditure reduction, medical care quality improvements and end user needs is required to attain and sustain a national patient health system diffusion with an interoperability model.

The United States government can benefit from our findings and observations by softening their stance on EMR system adoption. Instead of a position as a relatively authoritative policy-maker, maybe a position as a sincere orchestrator between the many stakeholders of the EMR innovation would be fruitful in terms of adoption. This requires acknowledging that the decision to diffuse electronic records is not held by the government or any one party. Rather, governments can work on converging the needs of all payer, provider, public and private groups, especially physicians, and bridging the gap between their needs and expectations. This benefit can also extend to other organizations, outside governments, who may hold that role of an orchestrator with unbiased interests in the adoption.

There is a need for increased education on EMR systems, their role, their benefit and the value they bring to the future of healthcare to facilitate and expedite adoption. Medical networks and educational institutions can benefit from this by taking ownership of developing enriched curriculums and training programmes for current and upcoming physicians. Physicians require knowledge and perceived industry benefits to form a positive attitude on an innovation. Had those two items been present and available, the pace to national diffusion and record sharing would have been faster. Physicians need greater awareness of the innovation and want to know what it is, what it aims to achieve and how the overall industry gains from it.

EMR system vendors have a practical advantage in knowing that the lack of financial benefit holds physicians back from supporting its adoption. Thus, vendors should work to keep costs of system setup and implementation manageable in order to attract adopters. Concurrently, they should develop and communicate the return-on-investment story and how adopting their EMR product will help practices gain relative advancement over competitors or improve their current situation. Financial institutions interested in healthcare investment may also facilitate access to funding for practices looking to implement EMR systems, to help manage the financial burden.

## Supplementary Information

**Additional file 1. **Survey questionnaire: the list of the final survey questions developed for this study along with their response options and codes.

**Additional file 2. **SAMPL guidelines checklist: list of the SAMPL guidelines related to hypothesis and regression testing with the respective location in the manuscript where the guideline is referenced or validated.

**Additional file 3. **Data file: Microsoft Excel file of the survey responses for the 13 questions and demographics for the 382 United States physicians.

## Data Availability

All data generated or analysed during this study are included in this published article and its additional information files.

## Abbreviations

AOM: Academy of Management
ARRA: American Recovery and Reinvestment Act
DOI: Diffusion of innovation
EMR: Electronic medical records
GEM: Grenoble Ecole de Management
SAMPL: Statistical Analyses and Methods in the Published Literature
SPSS: Statistical Package for the Social Sciences
TAM: Technology acceptance model
TPB: Theory of planned behaviour
TRA: Theory of reasoned action
