# Supplementary material for: Psychological, social and technical factors influencing electronic medical records systems adoption by United States physicians: a systematic model
Source: Health Res Policy Syst. 2022 May 2;20:48. doi: 10.1186/s12961-022-00851-0 (PMC9063322; doi:10.1186/s12961-022-00851-0)
Supplement: Supplementary file 1 — Additional file 1. Survey questionnaire: the list of the final survey questions developed for this study along with their response options and codes. [file 12961_2022_851_MOESM1_ESM.docx]

| **Survey Questionnaire** | | |
| --- | --- | --- |
| **Variable** | **Question** | **Response** |
| Intention | I intend to pursue or support the adoption and advancement of an electronic medical records system. | 1=Completely disagree  2=Disagree  3=Somewhat disagree  4=Neither agree nor disagree  5=Somewhat agree  6=Agree  7=Completely agree |
| Attitude | How would you like advancing the level of adoption and use of an electronic medical records system? | 1=Completely dislike  2=Dislike  3=Somewhat dislike  4=Neither like nor dislike  5=Somewhat like  6=Like  7=Completely like |
| Perceived Behavioral Control | How confident are you that your practice will succeed in advancing the adoption and use of an electronic medical records system? | 1=Completely unconfident  2=Unconfident  3=Somewhat unconfident  4=Neither confident nor unconfident  5=Somewhat confident  6=Confident  7=Complete confident |
| Peer Preference | How do your peer physicians, colleagues and medical affiliates feel about the adoption and use of an electronic medical records system? | 1=Completely dislike  2=Dislike  3=Somewhat dislike  4=Neither like nor dislike  5=Somewhat like  6=Like  7=Completely like |
| Government policy and Mandate | The federal government's policy and mandate are a primary consideration for advancing the adoption and use of an electronic medical records system in my practice. | 1=Completely disagree  2=Disagree  3=Somewhat disagree  4=Neither agree nor disagree  5=Somewhat agree  6=Agree  7=Completely agree |
| Industry Standards | Meeting medical industry standards is one of my primary considerations for advancing the adoption and use of an electronic medical records system in my practice. | 1=Completely disagree  2=Disagree  3=Somewhat disagree  4=Neither agree nor disagree  5=Somewhat agree  6=Agree  7=Completely agree |
| Knowledge | How knowledgeable are you about electronic medical records systems, their purpose, value, and requirements? | 1=Not at all  2=Slightly  3=Moderately knowledgeable  4=Very  5=Completely |
| Perceived Industry Benefits | The advancement of adoption and use of electronic medical records systems in medical practices will benefit health care in general. | 1=Completely disagree  2=Disagree  3=Somewhat disagree  4=Neither agree nor disagree  5=Somewhat agree  6=Agree  7=Completely agree |
| Perceived Usefulness | An advanced adoption of an electronic medical records system is useful to me. | 1=Completely disagree  2=Disagree  3=Somewhat disagree  4=Neither agree nor disagree  5=Somewhat agree  6=Agree  7=Completely agree |
| Perceived Ease of Use | Using the electronic medical records system in my practice is __________. | 1=Extremely difficult  2=Difficult  3=Somewhat difficult  4=Neither easy nor difficult  5=Somewhat easy  6=Easy  7=Extremely easy |
| Financial Ability | My practice has sufficient financial funding or subsidies to advance the adoption of an electronic medical records system. | 1=Completely disagree  2=Disagree  3=Somewhat disagree  4=Neither agree nor disagree  5=Somewhat agree  6=Agree  7=Completely agree |
| Workflow Benefits | With advancing the adoption and use of an electronic medical records system in my practice, the operations and workflow will become __________. | 1=Extremely harder  2=Harder  3=Somewhat harder  4Neither easier nor harder  5=Somewhat easier  6=Easier  7=Extremely easier |
| Relative Advancement | How advanced is the adoption and use of an electronic medical records system at your practice compared to other practices? | 1=Extremely less  2=Less  3=Somewhat less  4=Neither more nor less  5=Somewhat more  6=More  7=Extremely more |
