# Supplementary material for: Psychological, social and technical factors influencing electronic medical records systems adoption by United States physicians: a systematic model
Source: Health Res Policy Syst. 2022 May 2;20:48. doi: 10.1186/s12961-022-00851-0 (PMC9063322; doi:10.1186/s12961-022-00851-0)
Supplement: Supplementary file 2 — Additional file 2. SAMPL guidelines checklist: list of the SAMPL guidelines related to hypothesis and regression testing with the respective location in the manuscript where the guideline is referenced or validated. [file 12961_2022_851_MOESM2_ESM.docx]

| **SAMPL Checklist** | |
| --- | --- |
| **Guideline** | **Reference** |
| State the hypothesis being tested. | Methods section and Table 3 |
| Identify the variables in the analysis and summarize the data for each variable with the appropriate descriptive statistics. | Table 1 |
| If possible, identify the minimum difference considered to be clinically important. | N/A |
| For equivalence and non-inferiority studies, report the largest difference between groups that will still be accepted as indicating biological equivalence (the equivalence margin). | N/A |
| Identify the name of the test used in the analysis. Report whether the test was one- or two-tailed (justify the use of one-tailed tests) and for paired or independent samples. | Hypotheses and Model Testing subsection of Results, paragraph 1 |
| Confirm that the assumptions of the test were met by the data. | Hypotheses and Model Testing subsection of Results, paragraph 1 |
| Report the alpha level (e.g., 0.05) that defines statistical significance. | Table 2 |
| At least for primary outcomes, such as differences or agreement between groups, diagnostic sensitivity, and slopes of regression lines, report a measure of precision, such as the 95% confidence interval | Table 2 |
| Do NOT use the standard error of the mean (SE) to indicate the precision of an estimate. The SE is essentially a 68% confidence coefficient: use the 95% confidence coefficient instead. | Hypotheses and Model Testing subsection of Results and Table 2 |
| Although not preferred to confidence intervals, if desired, P values should be reported as equalities when possible and to one or two decimal places (e.g., P = 0.03 or 0.22 not as inequalities: e.g., P < 0.05). Do NOT report “NS”; give the actual P value. The smallest P value that need be reported is P <0.001, save in studies of genetic associations. | Results section including Table 2 |
| Report whether and how any adjustments were made for multiple statistical comparisons. | Results section |
| Name the statistical software package used in the analysis. | Hypotheses and Model Testing subsection of Results, paragraph 1 |
| Describe the purpose of the analysis. | Methods section |
| Identify the variables used in the analysis and summarize each with descriptive statistics. | Table 1 |
| Confirm that the assumptions of the analysis were met. For example, in linear regression indicate whether an analysis of residuals confirmed the assumptions of linearity. | Hypotheses and Model Testing subsection of Results |
| If relevant, report how any outlying values were treated in the analysis. | N/A |
| Report how any missing data were treated in the analyses. | Hypotheses and Model Testing subsection of Results |
| For either simple or multiple (multivariable) regression analyses, report the regression equation. | Results section |
| For multiple regression analyses: 1) report the alpha level used in the univariate analysis; 2) report whether the variables were assessed for a) colinearity and b) interaction; and 3) describe the variable selection process by which the final model was developed (e.g., forward-stepwise; best subset) | Results section |
| Report the regression coefficients (beta weights) of each explanatory variable and the associated confidence intervals and P values, preferably in a table. | Results section and Table 2 |
| Provide a measure of the model's "goodness-of-fit" to the data (the coefficient of determination, r 2, for simple regression and the coefficient of multiple determination, R 2 , for multiple regression) | Results section and Table 2 |
| Specify whether and how the model was validated. | Model Verification subsection or Results section |
|  |  |
